# Supplementary material for: 3D Printing Technology Based on Versatile Gelatin-Carrageenan Gel System for Drug Formulations
Source: Pharmaceutics. 2023 Apr 11;15(4):1218. doi: 10.3390/pharmaceutics15041218 (PMC10141357; doi:10.3390/pharmaceutics15041218)
Supplement: Supplementary file 1 [file pharmaceutics-15-01218-s001.zip › pharmaceutics-2276628-supplementary.pdf]

Table S1. Texture characteristics test results for different gel inks.

Gel ink	Hardness(gf)	Chewiness(gf)	Gumminess(gf)	Springiness	Cohesiveness
F1	0.8498	0.8210	0.8383	0.9793	0.9865
F2	1.5250	1.4459	1.5061	0.9600	0.9876
F3	2.4639	1.9786	2.3416	0.8451	0.9505
F5	2.0155	1.9322	1.9727	0.9794	0.9787
F6	2.0923	1.6881	1.7197	0.9820	0.9481
F7	1.5237	1.4321	1.5001	0.9548	0.9845
